# Supplementary material for: CmNAC73 Mediates the Formation of Green Color in Chrysanthemum Flowers by Directly Activating the Expression of Chlorophyll Biosynthesis Genes HEMA1 and CRD1
Source: Genes (Basel). 2021 May 8;12(5):704. doi: 10.3390/genes12050704 (PMC8151904; doi:10.3390/genes12050704)
Supplement: Supplementary file 1 [file genes-12-00704-s001.zip › genes-1207454-supplementary/Supplementary materials/Supplementary Figures.pdf]

Supplementary Materials

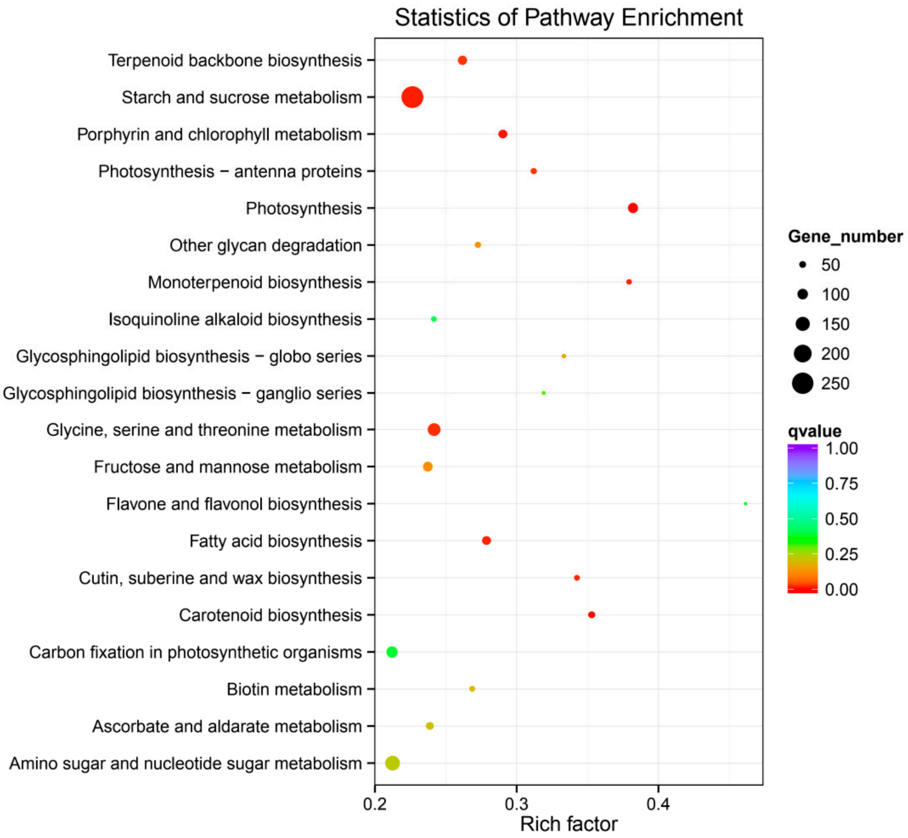

Figure S1. KEGG enrichment analysis of genes upregulated in *Chrysanthemum morifolium* cultivar 'Green anna' (AN) and *Chrysanthemum vestitum* (CV). Different colors indicate the q-value, and the circle size indicates the number of genes.

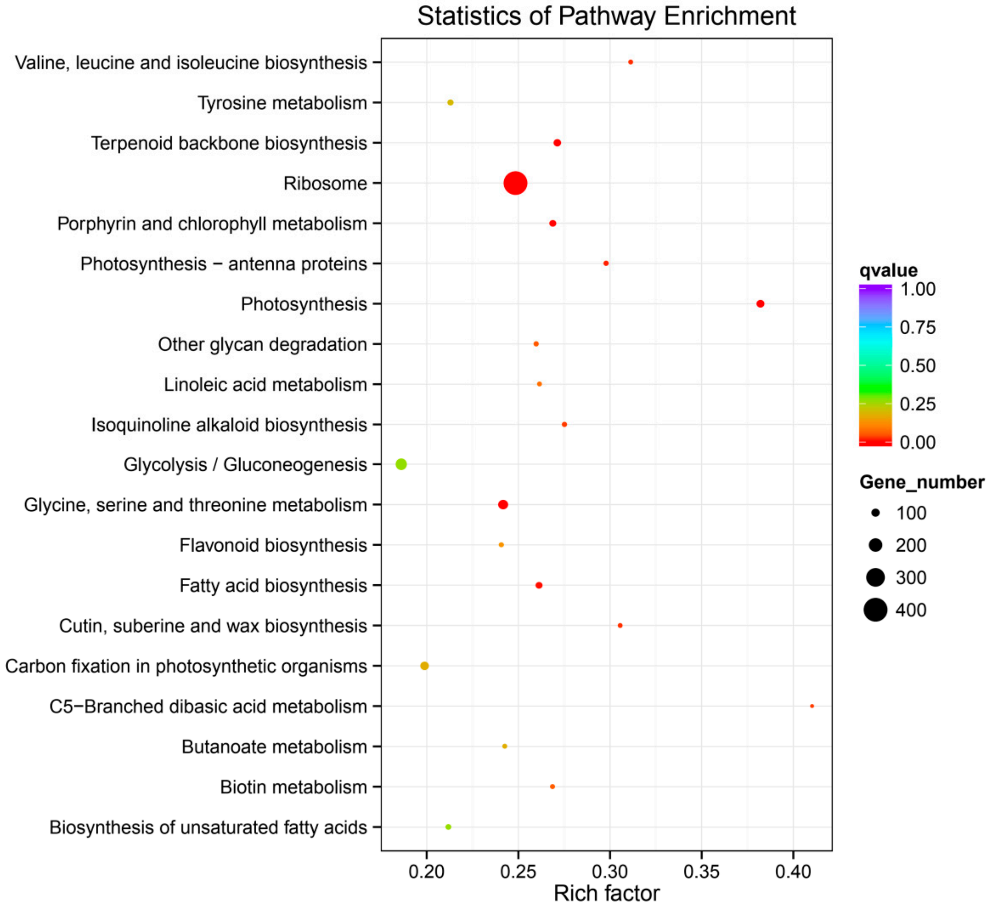

Figure S2. KEGG enrichment analysis of genes upregulated in *C. morifolium* cultivar 'Chunxiao' (CX) and *C. vestitum* (CV). Different colors indicate the q-value, and the circle size indicates the number of genes.

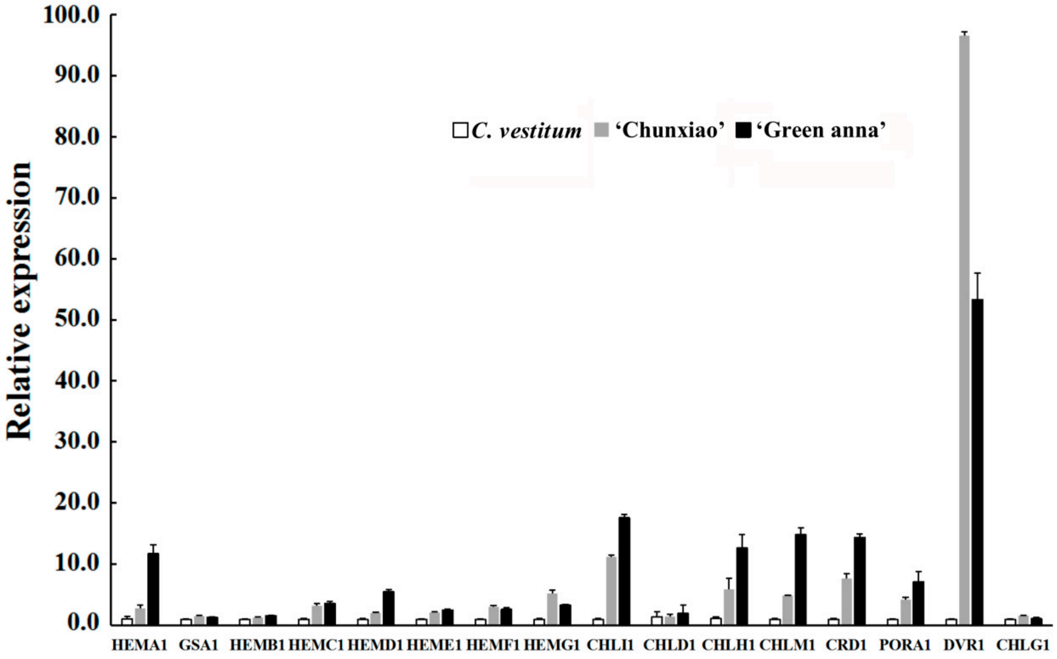

Figure S3. Expression analysis of chlorophyll synthesis-related genes in *C. vestitum* and *C. morifolium* cultivars 'Chunxiao' and 'Green anna' by quantitative real-time PCR (qRT-PCR). Data represent mean ± standard error (SE) of three biological replicates.

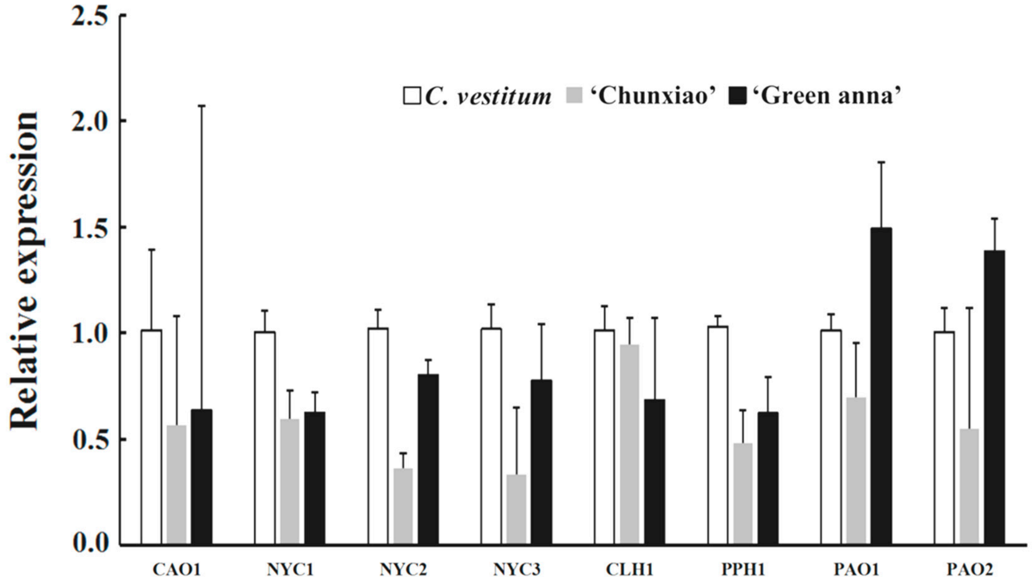

Figure S4. Expression analysis of chlorophyll cycling- and degradation-related genes in *C. vestitum* and *C. morifolium* cultivars 'Chunxiao' and 'Green anna' by qRT-PCR. Data represent mean ± SE of three biological replicates.

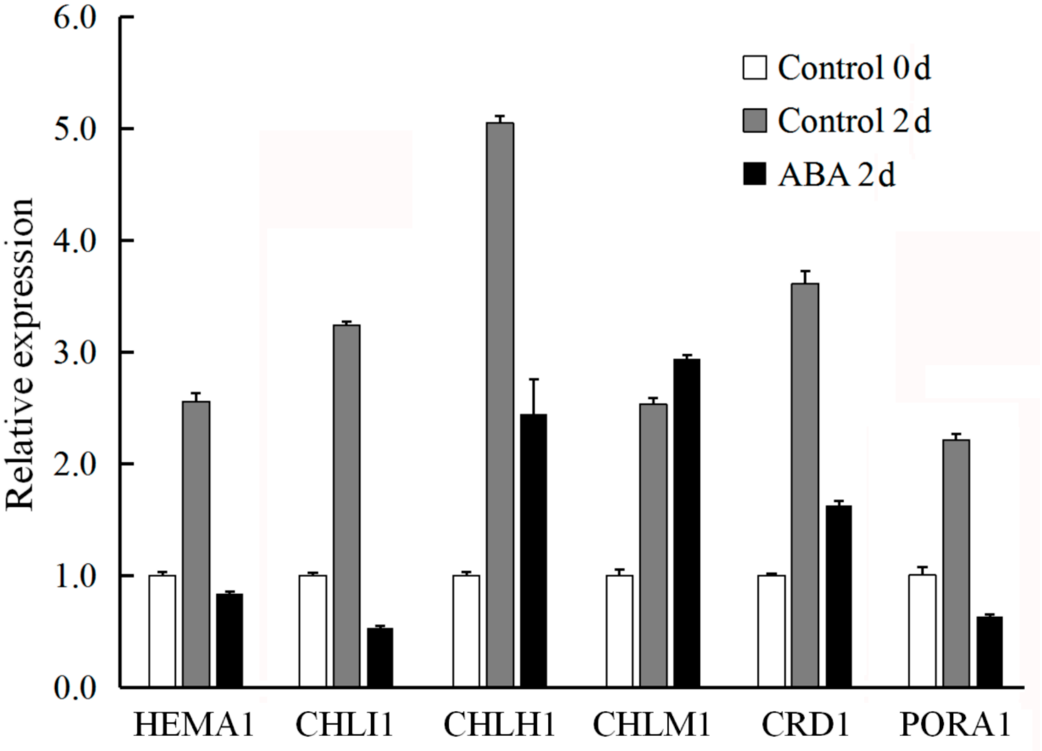

Figure S5. Expression analysis of chlorophyll synthesis-related genes in *C. morifolium* cultivar 'Lv Dingdang' treated with 20mg/L abscisic acid (ABA). Data represent mean ± SE of three biological replicates.

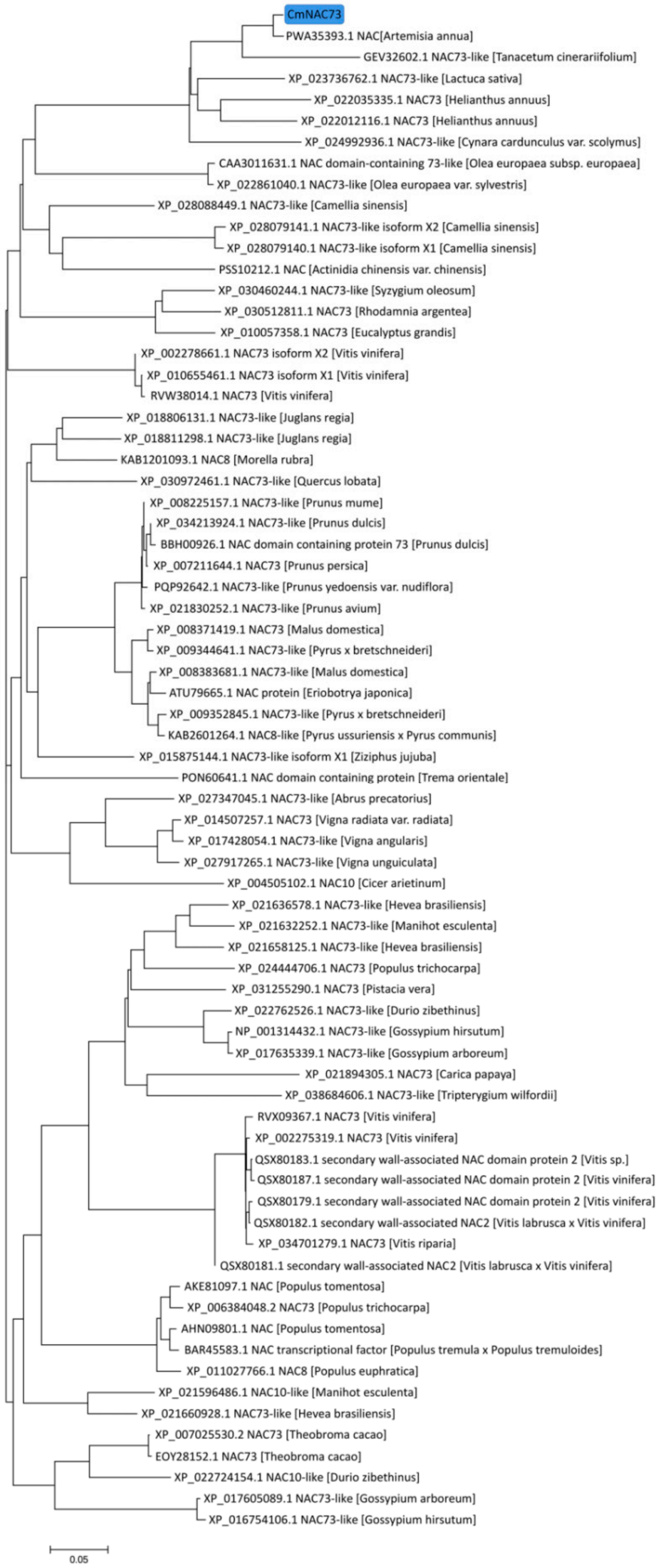

Figure S6. Phylogenetic analysis of CmNAC73 and other NAC proteins. Amino acid sequences of CmNAC73 and 71 NAC proteins from other plant species were aligned.
